# Modulated in vitro lymphocytes in the treatment of alcoholism: Experimental study

**DOI:** 10.1192/j.eurpsy.2021.2170

**Published:** 2021-08-13

**Authors:** E. Markova, I. Savkin

**Affiliations:** Neuroimmunology Lab, State Research Institute of Fundamental and Clinical Immunology, Novosibirsk, Russian Federation

**Keywords:** Cell technologies, modulated lymphocytes, alcoholism

## Abstract

**Introduction:**

Immune cells are dysfunctional during long-term ethanol consumption and may contribute to the progression from healthy to problem drinking. Lymphocytes from mice with chronic ethanol exposure characterized by impaired functional activity, manifested in the combination of increased spontaneous proliferation against the background of low sensitivity to T- cell mitogens.

**Objectives:**

We first demonstrated that original compound meta-chloro-benzhydryl-urea (m-ch-BHU) in vitro restored long-term alcoholized mice lymphocytes activity through GABA(A) receptors. We also revealed the possibility of animal’s behavioral regulation by the transplantation of immune cells with definite functional characteristics, also modulated by psychoactive drugs. Based on the previous results we investigated effects of m-ch-BHU modulated lymphocytes transplantation in recipients with experimental alcoholism.

**Methods:**

Male (CBAxC57Bl/6)F1 mice with 6-month 10% ethanol exposure were undergoing the transplantation of syngeneic long-term alcoholized mice lymphocytes, pretreated in vitro with m-ch-BHU. Recipient’s ethanol consumption, nervous and immune systems functional activities were estimated.

**Results:**

It was shown that transplantation of lymphocytes with in vitro m-ch-BHU modulated functional activity caused in syngeneic recipients with chronic alcohol exposure essential ethanol consumption decrease and stimulation of motor and exploratory activities in the “open field” against the background of cytokines modulation in brain. The significant stimulation of humoral immune response, estimated by the relative number of antibody-forming spleen cells, and stimulation of DTH reaction were also detected in recipients after lymphocytes transplantation.

**Conclusions:**

Results demonstrated that transplantation of m-ch-BHU modulated lymphocytes caused positive psychoneuroimmunomodulating effect in animals with chronic alcohol exposure, so, it may be considered as a promising method in the treatment of alcoholism

**Disclosure:**

No significant relationships.

